# CD133 Positive Embryonal Rhabdomyosarcoma Stem-Like Cell Population Is Enriched in Rhabdospheres

**DOI:** 10.1371/journal.pone.0019506

**Published:** 2011-05-13

**Authors:** Dagmar Walter, Sampoorna Satheesha, Patrick Albrecht, Beat C. Bornhauser, Valentina D'Alessandro, Susanne M. Oesch, Hubert Rehrauer, Ivo Leuschner, Ewa Koscielniak, Carole Gengler, Holger Moch, Michele Bernasconi, Felix K. Niggli, Beat W. Schäfer, Part of this study was conducted in cooperation with the CWS Study Group

**Affiliations:** 1 Department of Oncology and Children's Research Center, University Children's Hospital, Zurich, Switzerland; 2 Roche Pharma Schweiz, Basel, Switzerland; 3 Functional Genomics Center, University of Zurich, Zurich, Switzerland; 4 Department of Pathology, University of Kiel, Kiel, Germany; 5 Pediatrics 5 (Oncology, Hematology, Immunology), Olgahospital, Klinikum Stuttgart, Stuttgart, Germany; 6 Department of Pathology, University Hospital, Zurich, Switzerland; Institute of Cancer Research: Royal Cancer Hospital, United Kingdom

## Abstract

Cancer stem cells (CSCs) have been identified in a number of solid tumors, but not yet in rhabdomyosarcoma (RMS), the most frequently occurring soft tissue tumor in childhood. Hence, the aim of this study was to identify and characterize a CSC population in RMS using a functional approach. We found that embryonal rhabdomyosarcoma (eRMS) cell lines can form rhabdomyosarcoma spheres (short rhabdospheres) in stem cell medium containing defined growth factors over several passages. Using an orthotopic xenograft model, we demonstrate that a 100 fold less sphere cells result in faster tumor growth compared to the adherent population suggesting that CSCs were enriched in the sphere population. Furthermore, stem cell genes such as *oct4*, *nanog*, *c-myc*, *pax3* and *sox2* are significantly upregulated in rhabdospheres which can be differentiated into multiple lineages such as adipocytes, myocytes and neuronal cells. Surprisingly, gene expression profiles indicate that rhabdospheres show more similarities with neuronal than with hematopoietic or mesenchymal stem cells. Analysis of these profiles identified the known CSC marker CD133 as one of the genes upregulated in rhabdospheres, both on RNA and protein levels. CD133^+^ sorted cells were subsequently shown to be more tumorigenic and more resistant to commonly used chemotherapeutics. Using a tissue microarray (TMA) of eRMS patients, we found that high expression of CD133 correlates with poor overall survival. Hence, CD133 could be a prognostic marker for eRMS. These experiments indicate that a CD133^+^ CSC population can be enriched from eRMS which might help to develop novel targeted therapies against this pediatric tumor.

## Introduction

The cancer stem cell hypothesis suggests that a small subpopulation of cells sharing common characteristics with normal stem cells (SCs) - such as capacity to self renew, potential to differentiate, extensive proliferation *in vivo*, and resistance to chemotherapeutics - is responsible for tumor development [1] and that tumors are organized hierarchically. This concept was first established in acute myeloid leukemia (AML) [2] and subsequently also in a number of solid tumors such as breast cancer where a CD44^+^/CD24^−/low^ CSC population was identified [3], in brain tumors [4], colon cancer [5], and melanomas [6]. Additionally, CSCs are postulated to be more resistant to standard chemotherapy [7], [8], [9], [10], [11] and might be responsible for tumor recurrence usually observed in the clinics. However, the concept is still controversial and the frequency of CSCs might vary between tumor entities. Some tumors might not be hierarchically organized at all. Therefore, the existence of such a cellular subpopulation most likely has to be established for each tumor type [12].

Rhabdomyosarcoma (RMS) is the most common soft tissue tumor in childhood representing 5 to 8% of all pediatric malignancies [13]. RMS is a member of the small blue round cell tumors additionally comprised of neuroblastoma, non-Hodgkin's lymphoma, Ewing's sarcoma and Wilm's tumor [14]. It occurs in most parts of the body, but more frequent sites are spaces surrounding the brain, the trunk and genitourinary tract [15]. It has been suggested that mesenchymal stem cells (MSCs) might be the origin of rhabdomyosarcomas and accordingly the origin of a potential rhabomyosarcoma stem cell might also be a mesenchymal one [16], [17]. However, some reports indicate that also neuronal cells can transform into malignant myogenic cells after activation and a large number of neuronal genes are expressed in RMS. Hence the origin of potential RMS stem cells remains to be determined [18], [19].

CD133, also known as Prominin1, is a five transmembrane protein with eight potential N-glycosylation sites. It was first described in murine neuroepithelial cells and was recognized as a human hematopoietic SC marker, because hematopoietic CD34^+^ progenitor cells express CD133 [20]. CD133 has been suggested as CSC marker in brain tumors [21], breast [3], colon [22], pancreatic [23], liver [24], skin [25], prostate cancers [26] and Ewing's sarcoma [27]. Furthermore, CD133^+^ glioma stem cells are more resistant to chemotherapy and radiation than bulk and the CD133 negative population [8]. Moreover, CD133 downregulation induced differentiation in neuroblastoma cell lines and thus increased sensitivity to drug treatment [28]. Therefore, CD133 could by itself also represent a potential marker for targeted therapy. Nevertheless, CD133 positive CSC populations in melanoma and prostate cancer are still controversially discussed [29], [30].

Here, we enriched for a CSC population in rhabdosphere cultures which are 100 fold more tumorigenic than adherent cells in xenograft experiments. This subpopulation expressed the stem cell genes *sox2*, *oct4*, *nanog*, *c-myc* and *pax3* to significantly higher levels and retains the capability to differentiate into adipocytes, myocytes and neuronal cells. Furthermore, the known stem cell marker CD133 was upregulated in rhabdospheres. CD133^+^ cells characterize a subpopulation which is more tumorigenic and resistant to chemotherapy than the negative population. In addition, high CD133 expression in human eRMS samples correlated with a poor overall survival.

Thus, our study demonstrates for the first time that rhabdospheres can be formed from eRMS cells which are enriched in a CD133^+^ CSC population.

## Materials and Methods

### Cell culture methods

Rh36 (kindly provided by Peter Houghton (St Jude Children's Hospital, Memphis, TN, USA)), RD, U87MG and MRC5 (purchased from the American Type Culture collection (LGC Promochem, Molsheim Cedex, France)) and Ruch2 (established in house) were cultured in Dulbecco's modified Eagle medium containing 10% fetal calf serum (FCS).

Sphere cultures were derived from and enriched over several passages by seeding the cell lines in a defined serum free medium (SC medium) consisting of Neurobasal medium (Invitrogen) supplemented with 10ng/ml EGF (R&D Systems), 20ng/ml b-FGF (R&D Systems) and 2× B27 (10ml; Invitrogen) [31]. Adipogenesis was induced as described [32], [33]. Briefly, after preparing spheroids, cells were seeded into chamber slides and treated with or without 0.1% DMSO for 3 days. After 8 days in differentiation medium, containing 85nM insulin, 2nM triiodthyronine (T_3_) and 10% FCS, cells were stained with OilRedO (ThermoScientific) [34]. Neurogenesis and Myogenesis were assayed as described [35]. Briefly, cells were seeded into 6 well plates and treated with different concentrations of retinoic acid (RA; 1nM, 10nM, 300nM). After 24 days in differentiation medium containing RA and 0.5% FCS, cells were fixed in 4% paraformaldehyde (PFA) and stained for differentiation markers. Resistance to chemotherapeutics was tested by seeding 2000 cells in a 6-well plate 48 hours before treatment. The cells were treated twice a week with different concentrations of cisPlatin (Sigma; 10 µM and 50 µM) and Chlorambucil (Sigma; 6.45 µM). Twice a week, colonies were counted and documented. For visualizing the colonies, we stained them with crystal violet according to Franken, et al. [36].

### Immunofluorescence, immunohistochemistry and flow cytometry/sorting

For immunofluorescence staining, cells were fixed in 4% PFA and blocked in medium containing 10% FCS and 0.5% Triton. Cells were stained over night at 4°C for CD133 (1/100) (polyclonal antibody, Abcam), GFAP (1/300) (monoclonal antibody, R&D Systems), myogenin (1/2) (F5D; monoclonal antibody, Developmental Studies Hybridoma bank) and N-CAM (1/2) (5.1H11; Developmental Studies Hybridoma bank)). Alexa Fluor 488 or 594 (1/200) (Invitrogen) antibodies were used as secondary antibodies. All stainings were analyzed with an Axioskop2 mot plus fluorescence microscope (Zeiss). Xenograft tumors were embedded in paraffin, fixed and analyzed for H&E, Myogenin (1/20) (Myf4, monoclonal antibody, Novocastra Laboratories Ltd) and desmin (1/20) (monoclonal antibody; Dako) by immunohistochemistry. As secondary antibody a horseradish peroxidase (HRP) labeled rabbit anti-mouse antibody (Epitomics) was used. Stainings were visualized with the Refine DAB-Kit (Leica).

For flow cytometry, cells were trypsinized, washed and stained (1/10) with a fluorochrome labeled antibody (CD133/2-APC, Miltenyi). All samples were measured with a BDFACSCanto II flow cytometer (BD Bioscience) or MoFlo high speed cell sorter (DakoCytomation) and analyzed with the software FlowJo.

### Molecular methods

RNA was extracted using RNeasy Plus Mini Kits (Qiagen). Reverse transcription was carried out using the high-capacity cDNA reverse transcription kit (Applied Biosystems) according to the manufacturer's instructions. RNA and cDNA concentrations were measured with a Nanodrop ND1000 spectrometer. Quantitative Real-Time PCR was performed using validated TaqMan Gene Expression Assays (Applied Biosystems) for POU5F1/OCT3-4 (Hs02397400_g1), NANOG (Hs02387400_g1), SOX2 (Hs01053049_s1), CMYC (Hs00153408_m1), PAX3 (Hs00992437_m1), NMYC (Hs00232074_m1), PROM1/CD133 (Hs01009261_m1) and GAPDH (Hs99999905_m1) as an endogenous housekeeping gene for normalization. Reactions were run using the standard conditions on an ABI 7900HT Fast Real-Time PCR machine. Relative fold difference was calculated using the −ΔΔCt method. Gene expression profiling of different RNA samples from different sphere passages (early = passage 3; intermediate = passage 5 to 7; late = passage 10) and their corresponding adherent control was performed by an Affymetrix Exonmicroarray (HuEx-1_0-st-v2). The samples were analyzed with the Genespring10 and Ingenuity IPA software and compared with published data sets (hematopoietic (GSE2666), FM95 (GSE10435), embryonic skeletal myoblast (GSE3230), mesenchymal stem cells (GSE2248), embryonic stem cells (GSE9440), neuronal cells (GSE10691), glioblastoma cells and patient samples (GSE7181), neurospheres (GSE8049) and prostate cancer samples (GSE10832)). The correlation of the samples was analyzed with a script programmed in R (Functional Genomic Center Zurich).

### Xenograft experiments

Xenograft experiments were approved by the veterinary office of the Canton of Zurich.

Different amounts of adherent cells and their corresponding sphere cultures were injected intra muscularly into the right leg of NOD.CB17-*Prkdc^scid^*/J (NOD/Scid) and NOD.Cg-*Prkdc^scid^ Il2rg^tm1Wjl^*/SzJ (NSG) mice (The Jackson Laboratory) and tumor size was determined every 2 to 3 days by measuring two diameters (d_1_ and d_2_) in right angles of both legs with a calliper. Tumor volumes were calculated using the following formula V = [4/3 π ½(d_1_+d_2_)]_right leg_−[4/3 π ½(d_1_+d_2_)]_left leg_.

### Patient characteristics

76 eRMS patients, 43 male and 33 female patients, were included from the CWS95 study. The age of the patients at diagnosis varied from a few months to 22 years.

### Statistical analysis

For in vitro experiments, Student's t test was used on triplicates. P values of less than 0.05 were considered significant.

## Results

### Rhabdospheres are enriched with cancer stem-like cells

**Figure 1:**
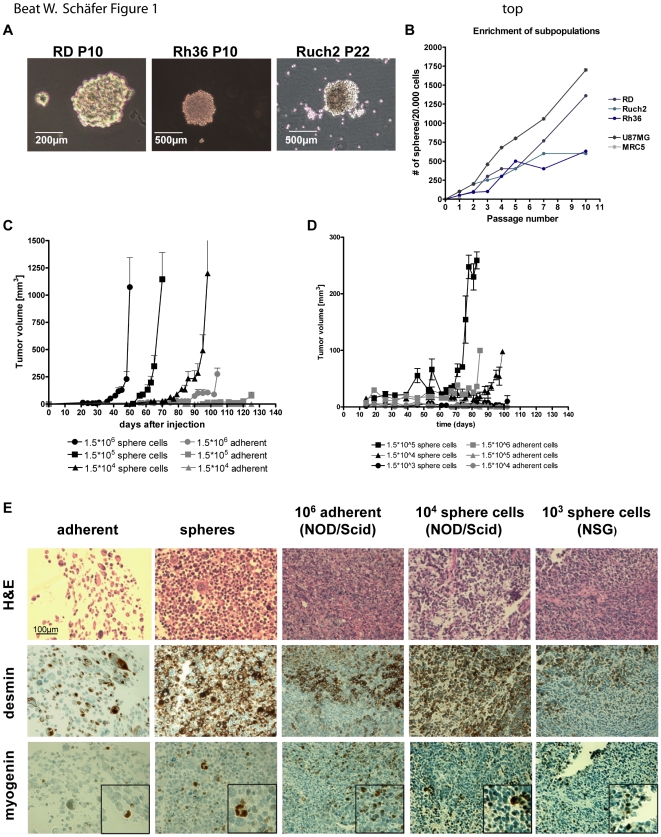
Cancer stem-like cells are enriched in Rhabdospheres. A), B) Embryonal rhabdomyosarcoma (eRMS) cell lines (RD, Rh36 and Ruch2) were cultured in stem cell medium (SC-medium) over several passages. A glioblastoma (U87MG) and a fibroblast (MRC5) cell line were used as controls. A) Representative phase contrast pictures of cultured RD, Ruch2 and Rh36 sphere cultures (400× magnification). B) Subpopulation enrichment over several passages (x-axis) was estimated by counting the obtained spheres per cell (y-axis). C), D) Limited dilution (10^6^, 10^5^ and 10^4^) of adherent versus sphere cells *in vivo*. Cells were intramuscularly (i.m.) injected into NOD/Scid (n = 6) (C) and NSG mice (n = 3) (D) at the indicated numbers and tumor growth (y-axis; tumor volume in mm^3^) was measured over time (x-axis). E) Immunohistochemical (IHC) stainings of xenograft tumor sections on a xenograft tissue microarray (TMA). Adherent and sphere cells were used as controls on the TMA. The TMA was stained for H&E and RMS markers (desmin and myogenin). Representative IHC stainings are shown (400× magnification). The small inserts represent magnifications of positively stained cells.

To determine whether RMS cells might contain a subpopulation of CSC cells, we attempted to grow embryonal rhabdomyosarcoma (eRMS) cell lines (RD, Rh36 and Ruch2) as rhabdomyosarcoma spheres (short rhabdospheres) in stem cell medium (SC-medium). A glioblastoma cell line (U87MG) and fibroblast cells (MRC5) were used as positive and negative controls, respectively. Three eRMS cell lines (RD, Rh36 and Ruch2) formed rhabdospheres under these conditions over several passages (Figure 1A). To test, whether sphere cells could be serially enriched, we seeded 20000 sphere cells over several passages into the SC-medium and determined the number of spheres at each passage (Figure 1B). Compared to the positive control U87MG sphere cultures which showed the highest enrichment over 10 passages (up to 1750 spheres per 20000 cells), 1300 spheres were counted for RD cultures after 10 passages, while Ruch2 and Rh36 sphere cultures could also be enriched albeit to a lesser extent (600 counted spheres) indicating that a subpopulation of cells with self renewal property can be enriched from three different eRMS cell lines. To investigate whether this self renewing subpopulation is more tumorigenic than the adherent population, we injected different numbers of RD cells and their corresponding sphere cultures (10^6^, 10^5^ and 10^4^) intramuscularly (i.m.) into the right leg of NOD/Scid mice (n = 6) and measured tumor growth over several weeks (Figure 1C). Xenograft tumors from sphere cultures started to grow around day 40 after injection, compared to adherent cells where we detected the earliest tumor growth around day 80 post injection. Moreover, tumor growth was observed when we injected 100 fold less sphere cells (10^4^), whereas no tumor growth was seen using the same number of adherent cells. 125 days after injection, in two out of six NOD/Scid mice injected with 10^5^ adherent cells, a small tumor was seen, while we detected tumors in every mouse injected with 10^5^ sphere cells already after 60 days. These results were subsequently confirmed in a second mouse strain, namely NSG mice, where tumor growth was observed with 10^5^, 10^4^ and 10^3^ (one out of three mice) injected sphere cells, but only with 10^6^ adherent cells (Figure 1D). Therefore, in both mouse models cells from sphere cultures are more tumorigenic and fewer cells are needed for tumor growth compared to adherent cells. To demonstrate that all xenograft tumors were indeed RMS tumors, we collected tumor samples and constructed a xenograft tissue microarray (TMA) with adherent RD cells and corresponding sphere cultures as controls. Stainings of the TMA with RMS markers, myogenin and desmin, was positive in both adherent cells and spheres (Figure 1E) which were negative for markers of other small blue round cell tumors (CD45, CD99, cytokeratin (CK), S100b, Synaptosin, smooth muscle actin (SMA) and WT1; data not shown). Furthermore, all xenograft tumors displayed typical RMS hallmarks such as multinucleated cells and positive stainings for desmin and myogenin, irrespective of the mouse strain they were grown in. These results confirmed that all xenograft tumors represented RMS tumors with similar features. We conclude from these experiments that a subpopulation of RMS cells can be enriched in sphere cultures over several passages which is more tumorigenic *in vivo* and therefore could represent a potential CSC population.

### Sphere cultures have stem cell characteristics

**Figure 2:**
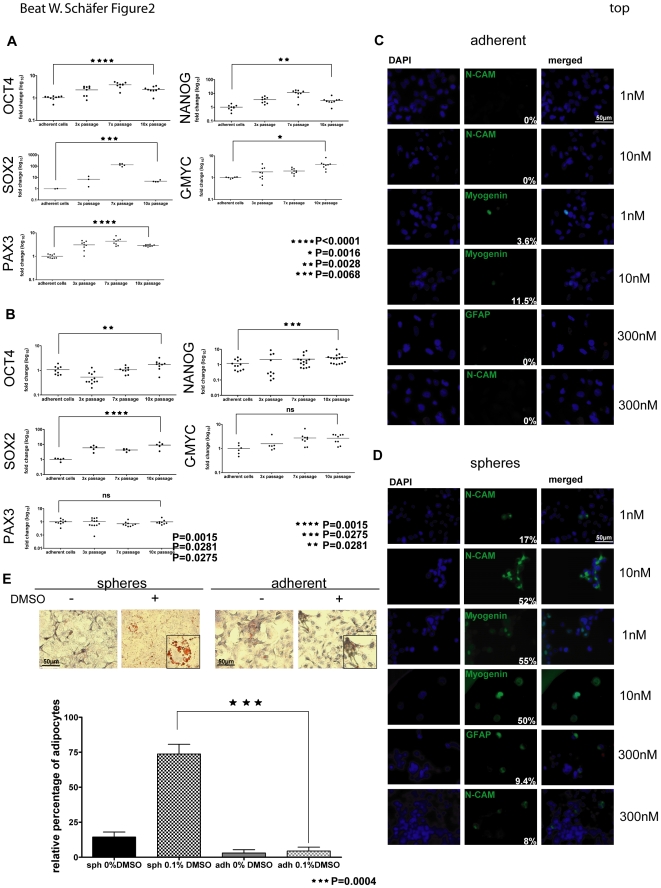
Sphere cultures have stem cell characteristics. A), B) Expression analysis of stem cell genes (*oct4*, *nanog*, *sox2*, *c-myc* and *pax3*) by Real-time PCR. RD (A) and Rh36 (B) adherent cells and 3 different sphere culture passages (3×, 7×, 10×) were compared. C), D) RD cells (C) and their corresponding sphere cultures (D) were treated with retinoic acid (1nM, 10nM, 300nM) for 24 days and stained for differentiation markers (N-CAM, myogenin and GFAP). Percentage of positivity was calculated by counting 3 different random microscopic fields with at least 30 cells. E) RD cells and spheres were treated with 0.1% DMSO for 3 days. After additional 8 days, cells were stained for OilRedO. Percentage of cells with fatty vacuoles was calculated by counting 4 independent slides. Representative pictures and magnifications (small box) of OilRedO stainings are shown. For A) ★★★★ P<0.0001; ★★ P = 0.0028; ★ P = 0.0016; ★★★ P = 0.0068. For B) ★★★★ P = 0.0015; ★★ P = 0.0281; ★★★ P = 0.0275. For E) ★★★ P = 0.0004. Abbreviations: ns, not significant; GFAP, glial fibrillary acidic protein; N-CAM, neural cell adhesion molecule; sph, spheres; adh, adherent; DMSO, dimethylsulfoxid.

To further substantiate the notion that rhabdospheres are enriched for CSC, we quantified the expression levels of several known SC genes like *oct4*, *nanog*, *c-myc*, *sox2* and *pax3* with real-time PCR in different passages of sphere cultures (passages 3, 7, 10) compared to adherent cells. While *oct4* and *pax3* showed the highest upregulation in RD sphere cultures (P<0.0001), also *c-myc* (P = 0.0016), *sox2* (P = 0.0068) and *nanog* (P = 0.0028) were significantly upregulated (Figure 2A). Similar results were obtained with Rh36 cells with the exception of *pax3* and *c-myc* which did not change significantly (Figure 2B). This could be due to already high endogenous expression levels in the adherent Rh36 cell line when compared to RD adherent cells (data not shown). Therefore, we selected RD cells for all subsequent experiments.

It has been shown that cells with multilineage differentiation potential can differentiate into neuronal cells, myocytes and adipocytes after treatment with dimethylsulfoxid (DMSO) or retinoic acid (RA) [32], [35], [37], [38]. On that basis, we next assessed to which extent RD cells can be differentiated towards these lineages. First, we treated adherent and sphere cultures with different concentrations of RA (1nM, 10nM and 300nM). After 24 days, cells were stained with myogenic (myogenin, N-CAM) and neuronal markers (GFAP, N-CAM) (Figure 2C, D). Although adherent cells expressed low levels of myogenin (3,6% and 11,5%) after treatment (Figure 2C), sphere cultures showed much stronger upregulation of myogenin positivity (∼50%) (Figure 2D). The highest expression of N-CAM (52%) was detectable after treatment with 10nM RA. While both spheres and adherent cells were negative for myogenin when treated with 300nM RA, we observed positive stainings for N-CAM and GFAP (9,4%), indicative of neuronal differentiation, only in sphere cultures (Figure 2D) and not in adherent cells (Figure 2C). In contrast, no GFAP positive cell was found after 1 and 10nM RA treatment (data not shown). Untreated controls were negative for all markers analyzed (data not shown).

To differentiate cells towards adipocytes, we treated spheroids from both adherent and sphere cells for 3 days with DMSO. After subsequent cultivation in appropriate differentiation medium for 8 days, around 5% of DMSO treated adherent cells were positive for fatty vacuoles (Figure 2E). However, sphere cultures had positively stained fatty vacuoles in up to 90% (mean 73.75%) of the cells when treated with DMSO (Figure 2E).

In conclusion, sphere cultures had a significantly increased expression level of stem cell genes and regained the capability to differentiate towards neurogenic, myogenic and adipogenic lineages with appropriate stimulants. These results indicate that stem-like cells are enriched in rhabdospheres.

### CD133 is upregulated in sphere cultures

To characterize sphere cultures in further detail and to identify marker proteins specifically up- or downregulated, a gene expression profiling was performed with a human exonmicroarray (HuEx-1_0-st-v2) for both RD and Rh36 cells and three different passages (early, intermediate and late) of their corresponding spheres.

**Figure 3:**
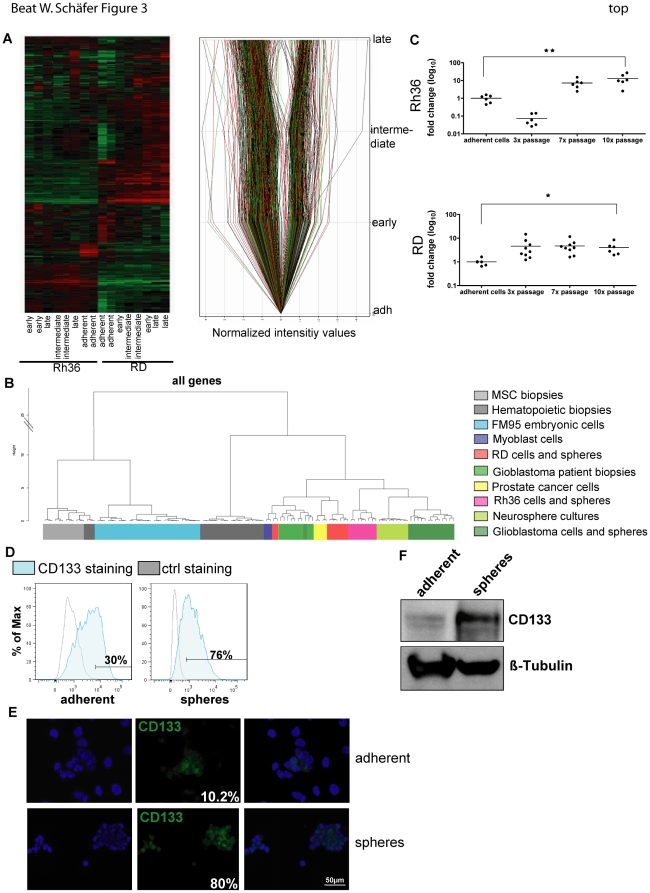
CD133 is upregulated in sphere cultures. Gene expression profiling (HuEx-1_0-st-v2) of two eRMS cells (RD and Rh36) and spheres (early, middle and late). A) left side: Cluster plot of RD and Rh36 cells and spheres. Right side: Analysis of RD samples. Genes, being up- or downregulated in RD spheres with a fold change of at least 2, are shown. B) Correlation plot of a metaanalysis performed with different publicly available expression data (hematopoietic and mesenchymal stem cells biopsies, FM95 cells, embryonic skeletal myoblast cells, embryonic stem cells, neuronal cells, glioblastoma spheres, cells and patient samples, neurospheres and prostate cancer samples) as indicated C) Expression of CD133 mRNA quantified by real-time PCR after correction with GAPDH levels as house-keeping gene. D) Flow cytometry analysis of CD133 (blue) expression. As controls unstained adherent and sphere cells were used, respectively (grey). E) Immunofluorescence staining of CD133 (green) of adherent and sphere cells. The nuclei were counterstained with DAPI (blue). Fields of two independent slides with at least 50 cells each were counted and the percentage of positive stained cells calculated. F) Western blot analysis of CD133 protein expression in adherent and sphere cells. ß-Tubulin was used as a loading control. A representative blot is shown. For C) ★ P = 0.0284; ★★ P = 0.0079. Abbreviations: ctrl, control; MSC, mesenchymal stem cells.

In Figure 3A, a heat map of all samples is shown which revealed that RD and Rh36 cells cluster with their corresponding sphere cells indicating that both cell lines are more different from each other than their different passages. Nevertheless, in total 2217 genes (upregulated 1568 genes, downregulated 649 genes) are differentially expressed in RD spheres compared to adherent cells with a fold change of at least two. To restrict the number of genes and to find potential markers characterizing the rhabdospheres, a metaanalysis with different microarray samples publicly available (hematopoietic, FM95, embryonic skeletal myoblast, mesenchymal stem cells, embryonic stem cells, neuronal cells, glioblastoma cells and patient samples, neurospheres and prostate cancer samples) was implemented. All RMS samples, both adherent and rhabdospheres (red (RD) and pink (Rh36)), clustered together with neuronal and glioblastoma cells and their spheres, and patient samples (depicted in green) (Figure 3B). Due to this observation, we searched for genes commonly up- or downregulated in RD and glioblastoma sphere cultures compared to their corresponding adherent cells with a fold change of at least two (Table 1). 31 genes were identified and further subgrouped according to their subcellular localization; membrane (8 genes), secreted (1 gene), endoplasmatic reticulum ER membrane (1 gene), golgi apparatus (1 gene), cytoplasm (12 genes) and nucleus (8 genes). In addition, 12 genes are commonly downregulated (membrane (6), secreted (2), cytoplasm (4)). To be able to identify and isolate a putative CSC population, we were interested mainly in membrane proteins of which we identified 14 genes. One obvious candidate gene in this list was CD133 or Prominin1 which is a well described SC and CSC marker. Therefore, we validated CD133 as a potential marker of rhabdospheres at the expression level by performing real-time PCR (Figure 3C). In sphere cultures of both RD and Rh36, CD133 expression was indeed significantly upregulated. To verify these results on protein level, RD cells and spheres were stained for CD133 and analyzed by flow cytometry (Figure 3D), fluorescence microscopy (Figure 3E) and western blotting (Figure 3F). In all experiments, CD133 is upregulated in rhabdospheres compared to adherent cells also on protein level.

**Table 1:** List of genes up- or downregulated at least two fold in RD rhabdospheres.

upregulated			downregulated	
Localization	Chosen Gene IDs	Gene Symbol	Chosen Gene IDs	Gene Symbol
**Membrane**	2535	FZD2	2674	GFRA1
	7976	FZD3	3778	KCNMA1
	8842	PROM1	4907	NT5E
	23554	TSPAN12	7010	TEK
	51678	MPP6	7057	THBS1
	55704	CCDC88A	23768	FLRT2
	57633	LRRN1		
	84216	TMEM117		
**Secreted**	255743	NPNT	4015	LOX
			7424	VEGFC
**Golgi apparatus**	22836	RHOBTB3		
**ER membrane**	80055	PGAP1		
**Cytoplasm**	2037	EPB41L2	3433	IFIT2
	3157	HMGCS1	3437	IFIT3
	4133	MAP2	9060	PAPSS2
	6860	SYT4	10231	RCAN2
	9315	C5orf13		
	9456	HOMER1		
	9735	KNTC1		
	54874	FNBP1L		
	55792	PCID2		
	56992	KIF15		
	91057	CCDC34		
	113263	GLCCI1		
**Nucleus**	7552	ZNF711		
	9735	KNTC1		
	10926	DBF4		
	55769	ZNF83		
	64105	CENPK		
	81931	ZNF93		
	84250	ANKRD32		
	90317	ZNF616		
	151648	SGOL1		

These experiments suggest that CD133^+^ cells , a known CSC marker, are enriched in rhabdospheres and CD133 might be a potential CSC marker in RMS.

### CD133^+^ RMS cells are more chemoresistant and tumorigenic

**Figure 4:**
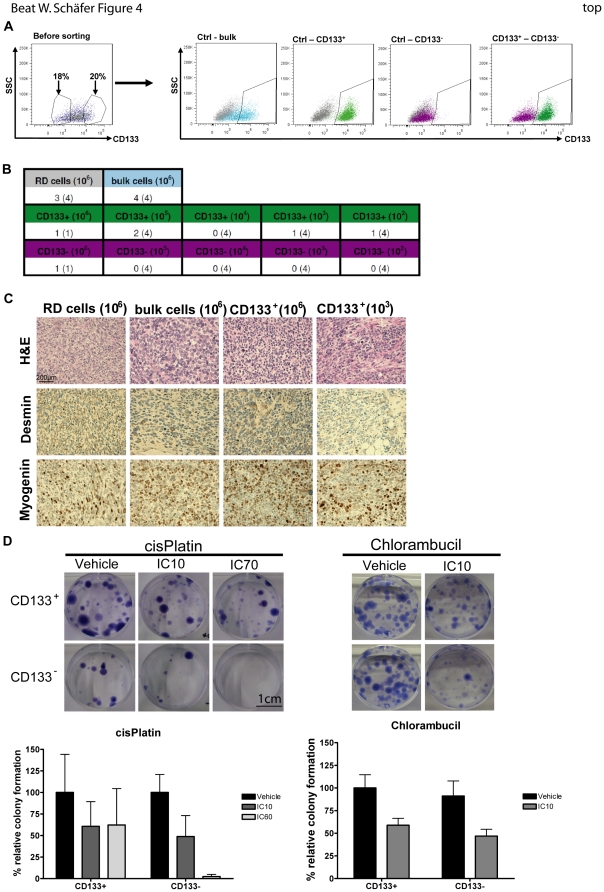
CD133^+^ RMS cells are more chemoresistant and tumorigenic. A) RD cells were stained for CD133 (blue) and sorted into CD133^+^ (green) and CD133^−^ (violet) populations with a MoFlo high speed cell sorter (DakoCytomation). Unstained RD cells were used as control (grey). After sorting the different fractions were reanalyzed by flow cytometry. B) Limited dilutions *in vivo* of different subpopulations (10^6^, 10^5^, 10^4^, 10^3^and 10^2^). Bulk stained (10^6^) and unstained cells without sorting (10^6^) were used as controls. Cells were injected i.m. into NOD/Scid mice (n = 4) and tumor growth measured. Numbers indicate mice with growing tumors. C) Immunohistochemical (IHC) analysis of all xenograft tumors (H&E, Myogenin and Desmin). Representative stainings are shown. D) Clonogenic assay with sorted subpopulations (CD133^+^ and CD133^−^). Cells were treated with cisPlatin (IC10 = 10 µM and IC60 = 50 µM) and Chlorambucil (IC10 = 6.45 µM). Colonies were visualized by crystal violet. cisPlatin: mean of 3 independent sortings ± SEM; Chlorambucil: mean of 2 independent sortings ± SEM. For D) ★★ P = 0.0377; ★ P = 0.0241. Abbreviations: IC, inhibitory concentration; ctrl, control.

To verify whether a CD133^+^ subpopulation is more tumorigenic and resistant to commonly used chemotherapeutics in RMS, we sorted RD cells for CD133 positive and negative (CD133^+^, CD133^−^) populations (Figure 4A) and performed limiting dilutions by orthotopical injections into NOD/Scid mice using adherent RD cells and unsorted bulk RD cells as controls. In contrast to the control where the highest number of cells injected (10^6^ cells) developed a tumor, mice injected with CD133^−^ cells (10^5^ – 10^2^) did not develop any tumor after 140 days. In contrast, we could detect at least one tumor in the CD133^+^ injected mice in three out of four dilutions (Figure 4B). To demonstrate that these tumors are indeed RMS tumors, we analyzed them by immunohistochemistry using known RMS markers as described before. All tumors were positive for desmin and myogenin and histologically identical with RMS tumors (Figure 4C). To investigate potential resistance to commonly used chemotherapeutics, we seeded sorted cells at low density 48 hours before starting treatment with cisPlatin and Chlorambucil. Cells were treated twice a week and colonies obtained were counted after staining with crystal violet (Figure 4D). CD133^+^ sorted RD cells were more resistant to treatment and formed viable colonies which developed significantly less in the CD133^−^ population.

Therefore, rhabdospheres are enriched for a CD133^+^ population being more tumorigenic and resistant to cisPlatin and Chlorambucil.

### High expression of CD133 correlates with poor overall survival

**Figure 5:**
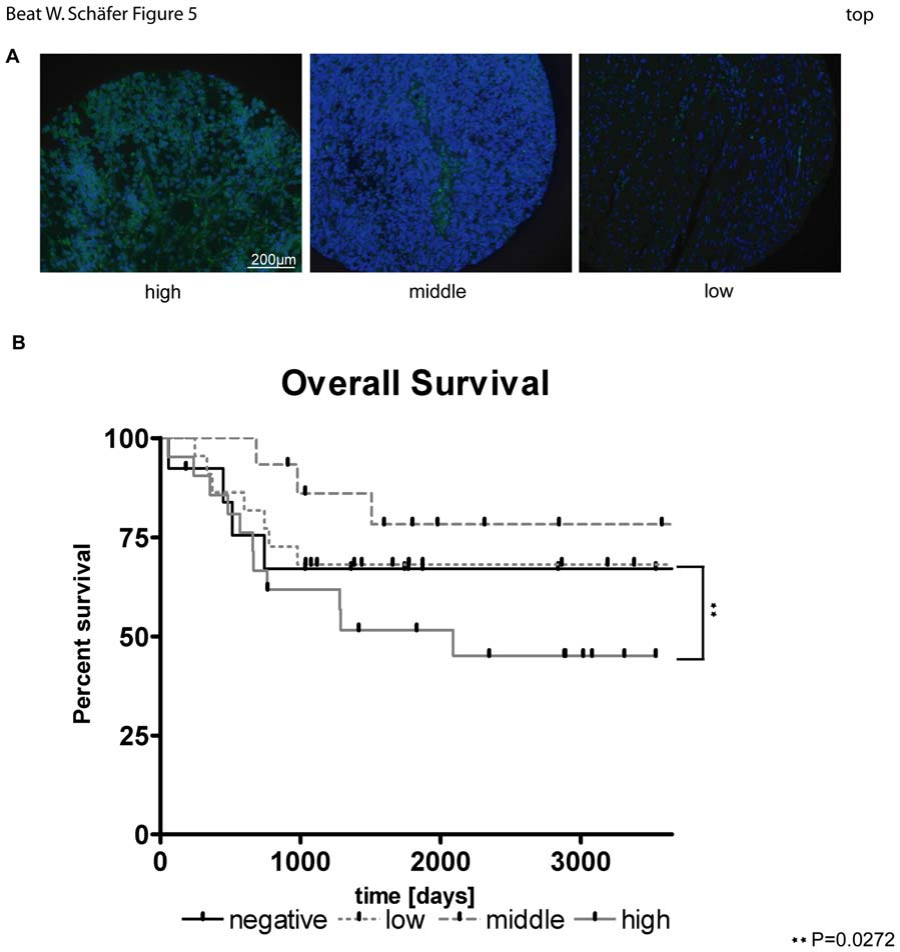
High expression of CD133 correlates with a poor survival rate. Immunofluorescence staining of a human RMS TMA with CD133 (green). Nuclei were counterstained with DAPI (blue). Two values were chosen for scoring: Staining intensity (1 = low, 2 = middle and 3 = bright) and number of positive cells (0 = 0; 1 = 1–10; 2 = 11–20; 3≥21). Both values were added up to the scorings negative (0 and 1), low (2 and 3), middle (4) and high (5 and 6). A) Stainings of representative tumor sections are shown for high, middle and low scorings. B) Overall survival of eRMS patients as shown by a Kaplan-Meier curve. For A) ★★ P = 0.0272.

Finally, we investigated whether a CD133^+^ subpopulation is also present in human patient material. To this end, we stained a human RMS TMA, first described by Wachtel [19], for CD133. For quantification, we scored for two variables, namely intensity of staining and number of positive cells. The added scores were used to classify the tumors as having negative, low, middle or high expression. ERMS patients showing no or low to intermediate CD133 expression showed an overall survival around 75% which is comparable with the survival rate of translocation negative RMS patients [39]. In contrast, patients with high expression of CD133 had a clearly worse survival (less than 50%, p = 0.0272, Figure 5A). Representative tumor sections of high, intermediate and low CD133 stainings are shown in Figure 5B.

These results therefore indicate that CD133 is a potential CSC marker in eRMS that might identify eRMS patients with a poor outcome.

## Discussion

Due to a better understanding of tumor organization, new treatment approaches that target directly a CSC population now seem possible [22]. It has been reported that not only leukemia [2] and carcinomas [3] have a subpopulation of cells with self renewal properties [27], but also some sarcomas such as Ewing's sarcoma might follow the cancer stem cell model [1]. For the most common sarcoma in childhood, RMS, no clear subpopulation has been identified until now [16], [17]. Therefore, we used a functional approach to investigate whether rhabdomyosarcoma tumors might have a subpopulation enriched in CSCs and are hierarchically organized.

We first adopted a sphere forming assay to enrich a subpopulation with stem cell properties *in vitro*. Testing different conditions of growth factor concentrations and media, sphere formation over several passages could be observed only in one condition which was described previously as a neuronal stem cell medium [31]. Several lines of evidence then indicate that these rhabdospheres are enriched for stem-like cells. First, limiting dilution in two different immunosuppressed mouse strains indicate that the rhabdosphere population is at least 100 fold more tumorigenic than adherent cells. In contrast, culturing cell lines representing the alveolar subtype of RMS in the same stem cell medium leads to formation of spheres which surprisingly were not tumorigenic after injection into immunosuppressed mice (data not shown). Hence, it seems unlikely that media conditions themselves were responsible for induction of the observed phenotypes and rather selection of a preexisting subpopulation was occurring specifically in eRMS. Second, our data analyzing expression levels of stem cell genes in rhabdospheres compared to adherent cells demonstrate that the stem cell genes *oct4*, *nanog*, *sox2*, *c-myc* as well as *pax3*, are significantly upregulated. While *sox2*, *nanog*, and *oct4* are required for induction of the pluripotent stem cell phenotype, *c-myc* expression also correlates with tumor formation and upregulation of this oncogene could trigger the higher tumor initiating potential [40]. *Pax3* is a known developmental marker expressed during muscle and brain development, repressed in adult tissue and connected to tumor formation and a poor overall survival [41], [42], [43].

As an additional hallmark of cancer stem cells [1], we investigated whether rhabdospheres have the potential to differentiate into multiple lineages. Indeed, rhabdospheres treated with DMSO and RA, respectively, differentiate towards adipogenic, myogenic and neurogenic lineages similar to what has been observed in cells with multilineage differentiation potential such as embryonal carcinoma cells [32], [33], [35]. These data support the concept that rhabdospheres contain cells with stem-like features and that RMS tumors are hierarchical organized [1].

Previous studies have suggested that mesenchymal stem cells could be the origin of RMS [17], [44], [45]. In contrast, our metaanalysis of exon microarray data with published data sets revealed that RMS samples had an expression profile more similar to neuronal cells and patients than mesenchymal stem cells. Furthermore, expression profiles also detected a large number of neuronal genes being expressed in RMS biopsies [19], [46], [47], [48] such as *pax3* which is crucial for the development of both the myogenic and neuronal lineage [42]. Interestingly, it has been demonstrated earlier that a population of myogenic, myf-5 positive cells can be derived from neural tube during mouse development [49]. These myf-5 positive cells co-express both neuronal and muscle markers, raising the intriguing possibility that the cell of origin of our CSC population could also be a multipotential stem cell derived from cells in the neuronal compartment. In support of this, it has also been described that neuronal stem cells can differentiate into malignant muscle cells after activation [18]. However, this issue needs to be addressed further in the future.

Previous studies have shown that CD133 marks hematopoietic stem cells [20] and cancer stem cells [29], in particular neuronal and mesenchymal CSCs [21]. Moreover, a CD133^+^ population was identified as a CSC population in sarcomas such as Ewing's sarcoma [27] and osteosarcoma [50] which was more resistant to chemotherapy and radiation [7], [8], [9], [10], [11]. It was therefore not surprising that CD133 emerged as a marker for RMS CSC in our study as well. Interestingly, also the fraction of CD133^+^ cells in both Ewing's sarcoma and RMS seem to be similar. It has been reported that expression of FGFR3 might mark a tumorigenic subpopulation in RMS. However, we did not find an increase in mRNA expression of this receptor in rhabdospheres (Figure S1). The same report also found that CD133 positive cells were not more tumorigenic than the negative population. However, the discrepancy with our study might be explained by the different CD133 epitopes that were used in the two studies. Here, using CD133 as a marker to sort cells which were then injected orthotopically into mice without prior cultivation in stem cell media, we readily detected tumor growth at lower cell numbers in CD133 positive versus CD133 negative cells. Indeed, in the CD133^+^ injected group one mouse at every dilution developed a RMS tumor which was not observed in the CD133^−^ population. The relatively low tumorigenicity detected in the sorted population in general is likely due to impaired viability of the cells by the sorting procedure. Interestingly, CD133^+^ sorted cells were also more resistant to cisPlatin and Chlorambucil treatment suggesting that anti-apoptotic or mismatch repair proteins are active. Indeed, we observed upregulation of several transcripts encoding mismatch repair proteins in rhabdospheres (data not shown).

Finally, we stained a human RMS tissue microarray (TMA) [19] for CD133 to demonstrate that a CD133^+^ population is also present in human tumor biopsies. Patients with high positivity for CD133 were found to have the worst overall survival, which could be explained by a higher recurrence. However, in a multivariate analysis using a cox regression model, we were not able to demonstrate that CD133 is an independent prognostic marker for eRMS since the number of patients in this group was too low. More patients will have to be included therefore in a future study. Nevertheless, CD133 might represent the first candidate marker to identify eRMS patients with poor survival and might be used to stratify patients in the future.

### Conclusion

Overall, our results demonstrate that cells with self renewal property that can drive tumorigenicity and have the potential to differentiate into multiple lineages are enriched in rhabdospheres. With CD133, we identified an already known CSC marker in an additional sarcoma [27], [50] whose expression also correlated with a poor prognosis in eRMS patients. Further characterization of this CD133 positive CSC population might lead to a better understanding of the development of RMS. It now seems possible to screen directly for therapeutically active substances targeting the CSC subpopulation in eRMS to further advance treatment of this childhood sarcoma.

## Supporting Information

Figure S1 **Prominin and fibroblast growth factor receptor (FGFR) expression in adherent and sphere cells.** A) Prominin1 and Prominin2 gene expression profiles in adherent and sphere cells analyzed by Genespring10 software. Intensity values were normalized to adherent cells. B) FGFR1, FGFR2, FGFR3 and FGFR4 gene expression profiles in adherent and sphere cells. Intensity values were normalized to adherent cells. C) Quantitative Real-time PCR with primers for FGFR3 (Hs00997400_g1) and for GAPDH was done with cDNA of adherent cells and three different passages of sphere cells. Quantitative results are indicated in arbitrary units (AU). FGFR3 was not differentially expressed in sphere cells compared to adherent cells.
(TIF)
