# Supplementary figures and images for: CD133 Positive Embryonal Rhabdomyosarcoma Stem-Like Cell Population Is Enriched in Rhabdospheres
Source: PLoS One. 2011 May 13;6(5):e19506. doi: 10.1371/journal.pone.0019506 (PMC3094354; doi:10.1371/journal.pone.0019506)

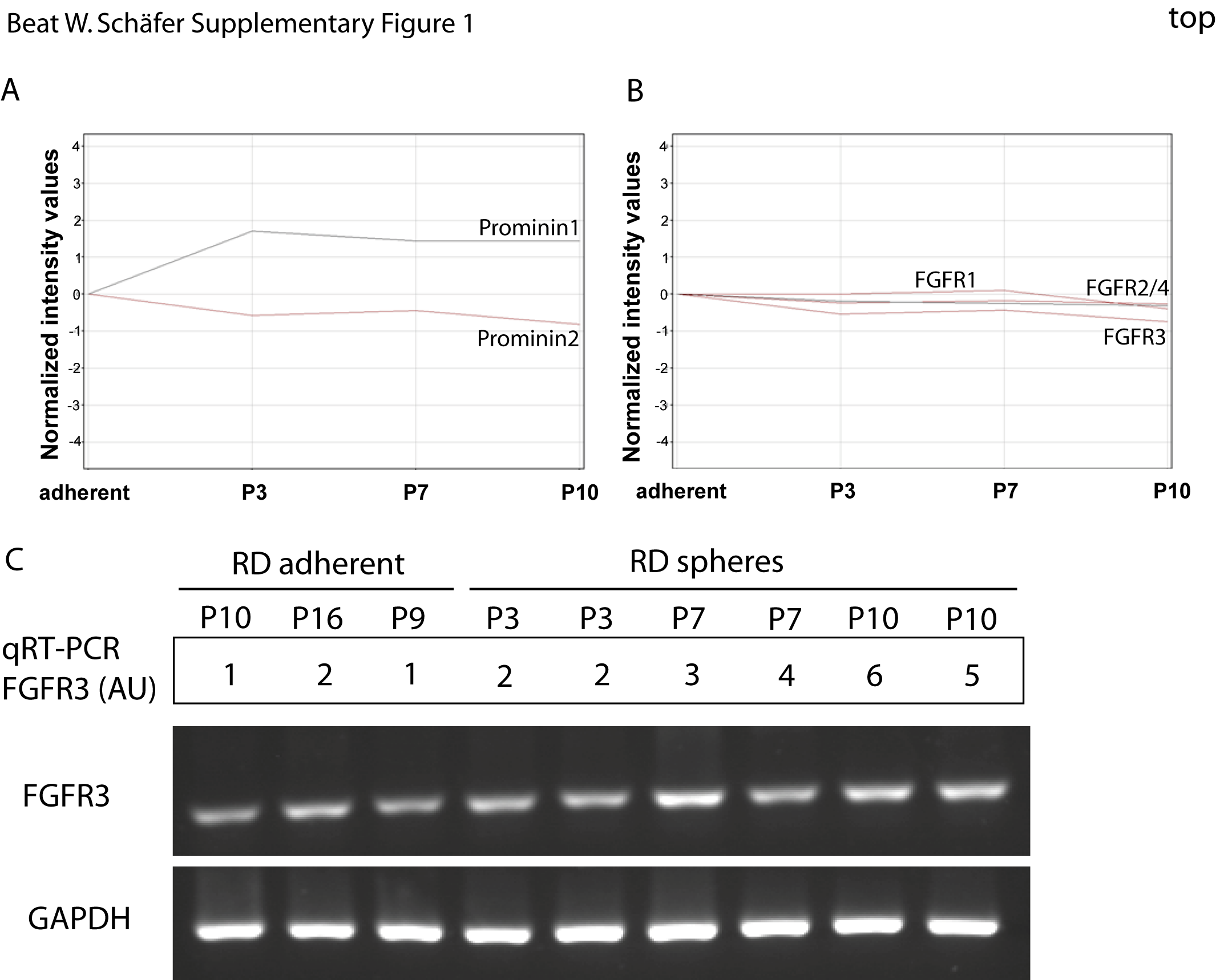

Supplement: Figure S1 — Prominin and fibroblast growth factor receptor (FGFR) expression in adherent and sphere cells. A) Prominin1 and Prominin2 gene expression profiles in adherent and sphere cells analyzed by Genespring10 software. Intensity values were normalized to adherent cells. B) FGFR1, FGFR2, FGFR3 and FGFR4 gene expression profiles in adherent and sphere cells. Intensity values were normalized to adherent cells. C) Quantitative Real-time PCR with primers for FGFR3 (Hs00997400_g1) and for GAPDH was done with cDNA of adherent cells and three different passages of sphere cells. Quantitative results are indicated in arbitrary units (AU). FGFR3 was not differentially expressed in sphere cells compared to adherent cells. (TIF) [file pone.0019506.s001.tif]
